# Where Are You Going, Nephrology? Considerations on Models of Care in an Evolving Discipline

**DOI:** 10.3390/jcm7080199

**Published:** 2018-08-03

**Authors:** Giorgina Barbara Piccoli, Conrad Breuer, Gianfranca Cabiddu, Angelo Testa, Christelle Jadeau, Giuliano Brunori

**Affiliations:** 1 Department of Clinical and Biological Sciences, University of Torino Italy, 10100 Torino, Italy; 2 Nephrologie, Centre Hospitalier Le Mans, 72000 Le Mans, France; 3 Direction, Centre Hospitalier Le Mans, 72000 Le Mans, France; cbreuer@ch-lemans.fr; 4 Nephrology, Brotzu Hospital, 09100 Cagliari, Italy; gianfranca.cabiddu@tin.it; 5 Association ECHO, 44000 Nantes, France; atesta@echo-sante.com; 6 Centre de Recherche Clinique, Centre Hospitalier Le Mans, 72000 Le Mans, France; cjadeau@ch-lemans.fr; 7 Nefrologia, Ospedale di Trento, 38100 Trento, Italy; gcbrunori@hotmail.com

**Keywords:** nephrology, health care, model of care, dialysis kidney transplantation, CKD, pre-dialysis care, prevention

## Abstract

Nephrology is a complex discipline, including care of kidney disease, dialysis, and transplantation. While in Europe, about 1:10 individuals is affected by chronic kidney disease (CKD), 1:1000 lives thanks to dialysis or transplantation, whose costs are as high as 2% of all the health care budget. Nephrology has important links with surgery, bioethics, cardiovascular and internal medicine, and is, not surprisingly, in a delicate balance between specialization and comprehensiveness, development and consolidation, cost constraints, and competition with internal medicine and other specialties. This paper proposes an interpretation of the different systems of nephrology care summarising the present choices into three not mutually exclusive main models (“scientific”, “pragmatic”, “holistic”, or “comprehensive”), and hypothesizing an “ideal-utopic” prevention-based fourth one. The so-called scientific model is built around kidney transplantation and care of glomerulonephritis and immunologic diseases, which probably pose the most important challenges in our discipline, but do not mirror the most common clinical problems. Conversely, the pragmatic one is built around dialysis (the most expensive and frequent mode of renal replacement therapy) and pre-dialysis treatment, focusing attention on the most common diseases, the holistic, or comprehensive, model comprehends both, and is integrated by several subspecialties, such as interventional nephrology, obstetric nephrology, and the ideal-utopic one is based upon prevention, and early care of common diseases. Each model has strength and weakness, which are commented to enhance discussion on the crucial issue of the philosophy of care behind its practical organization. Increased reflection and research on models of nephrology care is urgently needed if we wish to rise to the challenge of providing earlier and better care for older and more complex kidney patients with acute and chronic kidney diseases, with reduced budgets.

## 1. Introduction

Nephrology is the mother of all battles, at least in the eyes of nephrologists.

It is a rich, complex, multifaceted discipline offering, which is usually in exchange for long hours of hard work, the opportunity to practice in a hospital ward and witness the advances of organ transplantation, the challenges of internal medicine and the complexity of dialysis, the only “organ replacement therapy” that enables patients to live for decades on machine support.

Nephrologists today are facing the challenges that have come with new approaches to kidney transplantation and the treatment of glomerulonephritis. Their work involves exploring the genetic component of many so far unknown diseases. In some settings, elegant surgery for artero-venous fistulae and “interventional nephrology” are mainly practiced by nephrologists, while these subspecialties are increasingly practiced by surgeons, radiologists, or in intensive care [1,2,3,4,5].

The high prevalence of kidney diseases, affecting around 10% of the population worldwide, and the high cost of renal replacement therapy (RRT), consuming up to 2% of the health care expenditure for 1:1000 individuals (rounded prevalence of patients on RRT) are good reasons to explore the field of prevention and health care management [6,7,8,9,10,11]. Paediatric and geriatric nephrology have become self-standing subspecialties, while the field of obstetric nephrology is attracting increasing interest [12,13,14,15,16,17,18].

Nephrology played an important role in the development of bioethics: the first medical ethics committees were set up in Seattle, to choose from among clinically “fit” patients those who most “merited” to be treated from a social point of view, and it was this that led to the first profound changes in the United States (U.S.) health care system, allowing for dialysis treatment without economic restrictions [19,20,21]. Nephrology is still at the centre of bioethical discussions, as we debate how the trade in human organs should be dealt with, how palliative care should be defined and what its limits are, and how to improve the uneven availability of renal replacement therapy [22,23,24].

A balance between specialization and comprehensiveness, development and consolidation, efficacy, efficiency, and excellence is not only difficult to attain, but it also varies between countries and between models of care [1,2,24,25,26].

At least in Europe, cost constraints on the one hand, and competition with internal medicine and other specialties on the other, are obliging us to consider what the actual core of our specialty is. In this setting, we hope that our reflection, which is mainly based upon Italian and French examples, on the account of the direct experience of the authors, will highlight the strengths and weaknesses of different approaches to the organisation of care, which we tried to synthetize into four different models and ultimately help in identifying what road nephrologists should be taking.

### 1.1. Stars and Planets: Organizational Schemas

The complexity of nephrology eludes simplistic approaches.

**Figure 1:**
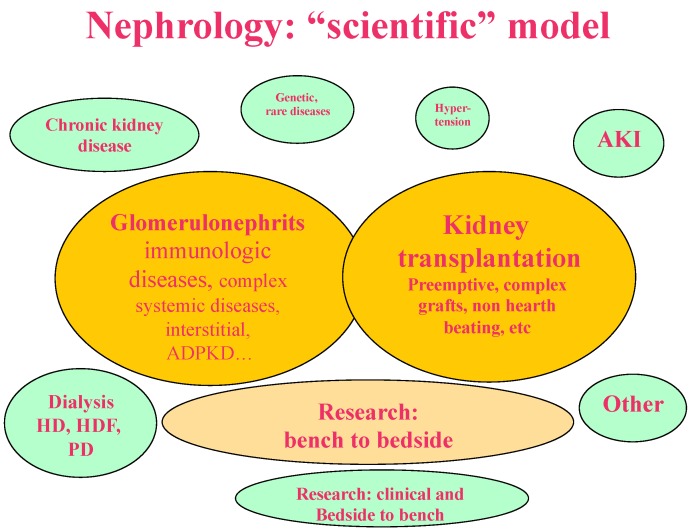
The “scientific” model: the focus is on glomerular diseases, and on kidney transplantation. Basic and bench to bedside research is highly developed.

**Figure 2:**
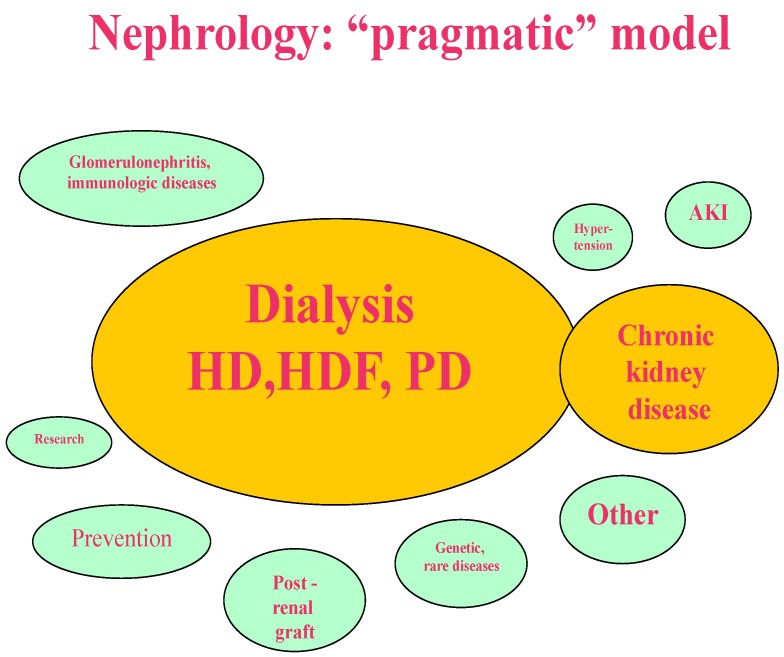
The “pragmatic” model: the focus is on dialysis and pre-dialysis care. Attention is on the most severe and common kidney diseases; research development is an option.

**Figure 3:**
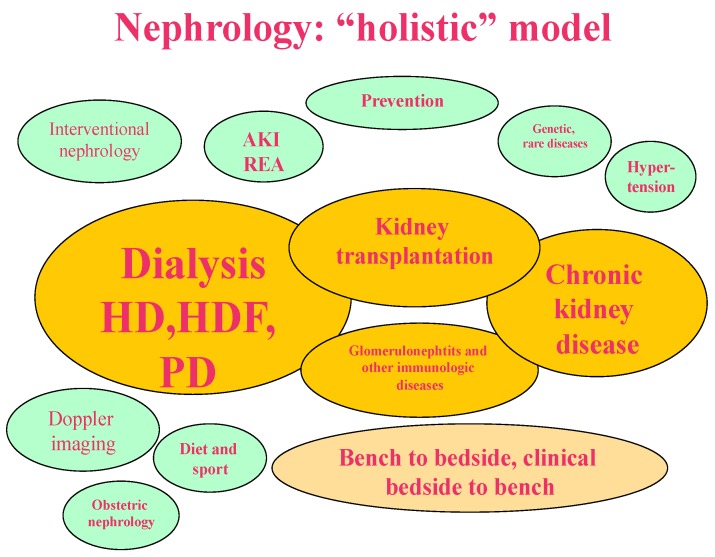
The “holistic” or “comprehensive” model: the focus is on a comprehensive system, in which all aspects of care and research are included.

**Figure 4:**
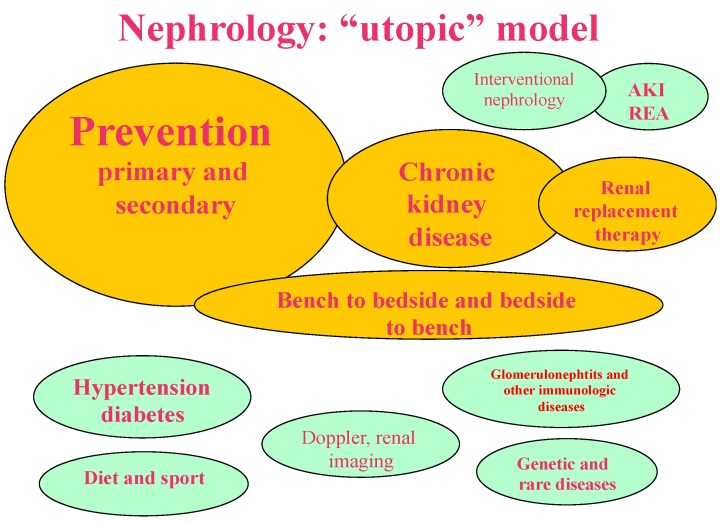
The “ideal-utopic” model: the focus is on prevention, that should decrease the need for chronic kidney disease (CKD) care and for renal replacement therapy. Research is highly developed, both ways, bench to bedside and bedside to bench.

The models depicted in Figure 1, Figure 2, Figure 3 and Figure 4 synthesize and schematize the three main options that are currently available (i.e., “scientific”, “pragmatic” and “comprehensive”), and hypothesize an “ideal” prevention-based approach (Figure 1, Figure 2, Figure 3 and Figure 4). These models are not mutually exclusive. They are often complementary (for example, the “scientific” and the “pragmatic” models), or develop in line with the availability of resources and the economic constraints of each context (public, private for profit, private non-profit). Furthermore, as usual, the real life is more faceted, and one model may merge into another; however, we felt that this attempt to classify them could ease the discussion, and possibly enhance our chances to develop an “ideal-utopic” one, based on prevention instead of on (late) action.

The aim of our analysis of these approaches is to enable us to reflect on the organizational aspects of nephrology, a discipline that, despite the great improvements that have been made and its enormous potential, fails to fully respond to patients’ needs, as witnessed by the significant number of cases that yearly reach end-stage kidney disease (up to 40% in some European countries), in which chronic kidney diseases developed without the nephrology care that could have retarded dialysis start and perhaps prolonged life [27,28,29,30].

Defending or expanding the nephrology workforce is a choice that clashes with the current European trend to replace specialist care services with large medicine wards, which are considered to be better able to respond to the needs of an ageing population with multiple comorbidity: greater awareness of nephrology’s aims and compass, and of its intervention models, can serve as a tool for optimizing this discipline’s resources.

### 1.2. The “Scientific Model”

We chose this definition to synthetize a model in which nephrology mainly focuses on potentially treatable diseases (glomerular, immunologic, systemic), and on kidney transplantation. The term “scientific” underlines the fact that this model is mainly found in University settings, where it is usually coupled with research activities. Likewise, transplantation, whose research potential remains enormous, requires multidisciplinary organization (intensive care, vascular surgery, urology, nephrology), which is more often found in university settings, and in large structures (Figure 1). Of course, this, as well as the other definitions, is a simplification of the more faceted situation, in which also other diseases, such as hereditary kidney ones (including polycystic kidney disease), or complex multiorgan disorders, are a matter for active “scientific” research. Care of these diseases needs a network, including diagnostic (genetic) and therapeutic approaches, which are more easily found in large University settings.

In this model, which is still widespread in university settings in Italy and France, predialysis and dialysis care usually play a minor role and dialysis care is mainly targeted to acute situations or at start of treatment. In France, since transplantation is only performed in university centres, the “scientific” structure is usually coupled with research units, promoting laboratory and bench-to-bedside research; chronic dialysis is usually mainly developed by associated non-profit private structures (“associations”), which also provide out-of-hospital care [31]. These choices have some important advantages: focusing clinical and research resources on potentially treatable kidney diseases and on transplantation (coupled with a highly efficient network of intensive care units) is probably a key reason for the high development of kidney transplantation in France [32].

Conversely, the most common diseases causing end stage kidney failure, as well as advanced chronic kidney disease, i.e., diabetes and hypertension, may be disregarded, losing an occasion for secondary prevention or at least for retarding the need for renal replacement therapy. Furthermore, leaving pre-dialysis and dialysis care out of the core studies of the country’s large university units may have critically reduced the exposure of students and trainees to fields such as peritoneal dialysis, nutritional, or pre-dialysis care of chronic kidney disease (CKD) patients, thus limiting the potential of out-of hospital care and integrated nutritional follow-up [32,33,34,35].

### 1.3. The “Pragmatic Model”

We chose this definition to identify a model in which nephrology care mainly focuses on dialysis, also, or mainly, on the account of economic reasons. Dialysis is therefore the core of this model, and pre-dialysis care develops around it, which is mainly in order to prepare patients for dialysis start (Figure 2). Other activities, such as interventional nephrology and post-transplant, or even care for rare diseases, are ancillary to well-functioning dialysis activity [27,28,29,36,37,38,39]. The development of these latter activities mainly depends on the preferences of each group, but can be limited by a lack of hospitalization facilities.

This model in increasingly found for example in Italy, where many small public nephrology services increasingly depend on larger referral centres for a series of activities, such as kidney biopsy or interventional procedures, and in France where most French “associations” depend on a structured relationship with a public centre.

The advantage of focusing on dialysis, probably nephrology’s most “specific specificity “, which overall in Europe treats between 400 and 700 patients per million population, is clear. The goals of integrating the care of CKD are not only to prepare patients for dialysis and to orient them to the different treatments that are available in a cost-effective (and sometimes gain-effective) manner, but also to offer a continuum of care, particularly important in the transition phases between pre-dialysis and dialysis care [36,37,38,39,40,41,42,43].

However, in particular, in private settings, or under strong cost constraints, treatment choices may be affected by the reimbursement system, which is graded for levels of care in many countries. Indeed, reimbursement is often higher for in-hospital and lower for home-based treatments, a policy that tends to disincentivize home dialysis in all its forms [44,45,46,47,48].

The examples of the virtual absence of peritoneal dialysis in private Italian structures (versus an overall prevalence of about 10%, almost limited to public centres) and the lack of diffusion of peritoneal dialysis in France (6.4% at the last report of the REIN Registry—REIN: Renal Epidemiology and Information Network), demonstrate the entity of the problem. Virtuous exceptions can be found: the re-emergence of home haemodialysis in France, thanks to conjoint public-associative efforts, and the renewed use of peritoneal dialysis, advocated by some patients’ associations, despite its lower economic yield [49].

The importance of the incentive-disincentive mode is clear. For example, when Switzerland created an incentive-disincentive system to promote peritoneal dialysis, this rapidly influenced how often this treatment, which had previously been virtually abandoned, came to be utilized [50,51]. With a handful of exceptions, small centres and for-profit facilities are more dependent on reimbursement, while larger facilities and public units tend to be more flexible, and can reinvest in innovative treatments or in treatments in which the clinical or social advantages are penalized by lower reimbursement.

**Figure 5:**
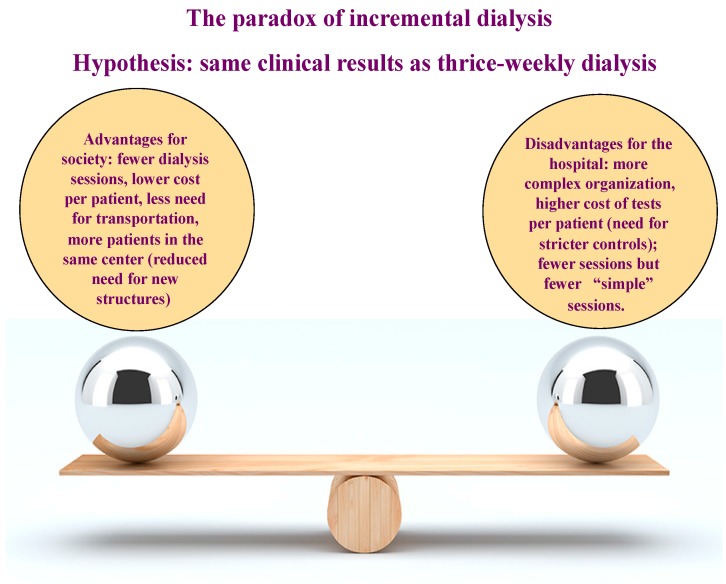
Paradoxes in care. The example of incremental dialysis. What is advantageous for the patient and for the society is not necessarily advantageous for the hospital.

The case for incremental dialysis is shown in Figure 5.

The basic idea of incremental dialysis is as logical as it is clinically sound: since in many cases, in particular in elderly patients, end-stage renal disease develops slowly, dialysis can be started at a “low dose” (for example, one exchange per day in peritoneal dialysis or a session once a week on haemodialysis), and the dose of dialysis can then be progressively increased in line with the decrease in residual kidney function. However, although in incremental dialysis there are both clinical benefits for patients (smoother dialysis start; progressive adaptation to treatment, reduced risk of iatrogenic effects) and financial advantages for the healthcare system (lower cost per patient, less need for transportation, less interference with patients’ work), problems may be created for the setting of care (need for frequent check-ups, more complex organization, lower exploitation of dialysis posts), so that this treatment may become less appealing from the financial point of view of single hospitals-institutions [23,52,53,54].

### 1.4. The “Holistic” or “Comprehensive Model”

We chose this definition, employing an overused term, holistic, and a pragmatic one, comprehensive, to identify a model in which nephrology is developed in all (or at least in as many as possible) of its possible branches.

Nephrology has a propensity for creating “hospitals within hospitals”, as it deals with chronic patients whose CKD modulates the natural history and the treatment of associated diseases and comorbidities (Figure 3).

Fragmenting the provision of care involves the obvious risks of keeping activities below the critical mass that is usually needed for excellence, increasing costs, and diverging practices. Conversely, it has the advantages of diversifying activities, facilitating recruitment, and updating nephrologists with polyvalent expertise, and, from the economic point of view, keeping more pathways open and integrating a range of occasional activities.

This is the model of the large, mainly (but not exclusively) university centres in Italy, in which a large group of physicians is responsible for virtually all aspects of care related to acute and chronic kidney diseases, including the treatment of hypertensive patients, of diabetic patients since the first signs of kidney involvement, of renal tubular disorders and kidney stones; in such a setting, nephrologists’ tasks may include vascular and peritoneal access, nutritional care, kidney biopsy, laboratory research, educational activities, university teaching, management of acute kidney diseases, all forms of chronic dialysis, and kidney transplantation since the immediate post-surgical phase. While only few of this plethora of activities are reflected in research, the flexibility of the system is probably at the basis of the substantial and varied body of articles that are published by the Italian Society of Nephrology.

International data indicate that the results of nephrology-related treatments (dialysis access, post-transplant care, interventional nephrology, diagnostic procedures, etc.) are at least as good when performed in nephrology as when they are delegated to other specialities [55,56,57,58,59,60]. While there is an obvious bias linked to the fact that only the most motivated and best-qualified centres perform these satellite activities, the evidence that is available indicates that this expertise should be maintained and developed when logistically feasible. One example is Italian nephrology’s contributions to the field of renal nutrition: despite the fact that in most settings nephrologists work without the support of a dietician (a specialist that relatively few Italian nephrology wards have), the development of the field at least equals that of other countries in which having a dietician on the staff is standard [61,62].

Similar considerations may apply to the management of acute kidney injury (AKI) in the intensive care Units.

The option of “multidisciplinary” units, with different specialists, may be a good compromise and a bridge towards other specialties, including oncology, in particular, for the emerging toxicities at the kidney level, hematology, for various diseases, including myeloma, amyloidosis and cryoglobulinaemia, urology for the management of patients with kidney stones, or of patients with reduced nephron mass, etcetera.

**Figure 6:**
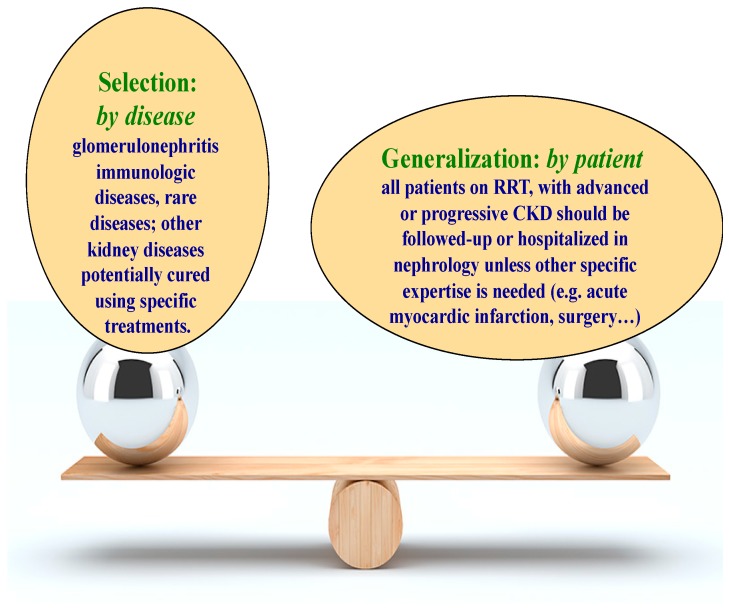
Paradoxes in care. The example of hospitalisation: should we focus on diseases or on patients?

The implications in terms of workload and workforce are evident but difficult to quantify in national and international comparisons. There is an urgent need for more research in this field, including an analysis of the results of similar procedures and treatments (including hospitalization, Figure 6) in nephrology units and other settings (for instance internal medicine), before pressure to reduce specialist activity leads to an irreversible loss of expertise in our discipline, as it occurred, for instance, for vascular access, whose expertise shifted from being mainly nephrologists’ to mainly a task of vascular surgeons, or for early stages of diabetic nephrology or hypertensive diseases, now mainly followed in internal medicine or endocrinology. The lack of efficiency of leaving these competences out of the Nephrology specialty are reflected by the lack of decrease not only of patients needing renal replacement therapy, but also of late referral of patients with end stage kidney diseases, even in these two contexts [28,29,36,37,38,39,40,41,42].

### 1.5. The “Ideal-Utopic Model”

We chose the terms ideal or utopic to identify a model of care based upon prevention, which is, in theory, the best approach in health care, hence the term “ideal”, but it may clash with the present organization of care, requiring a complete reorganization of the patient’s trajectory, not limited to renal diseases, hence the term “utopic”.

Preventing kidney diseases, slowing their evolution with timely treatment, and avoiding the need for renal replacement therapy is the ideal nephrology that should be strived for (Figure 4). Higher investment in prevention and early care would reduce the weight of severe chronic kidney disease, and, ultimately, the need for renal replacement therapy.

In this conceptual framework, the proverbial ounce of prevention is worth a pound of cure. A great deal of indirect evidence supports this model, advocated by most scientific associations [63,64,65,66,67]. Early referral, timely diagnosis, life-style changes, and education have all been linked to slower CKD progression and improved survival [28,29,36,37,38,39,40,41,42,68,69,70,71,72,73].

In this setting, a priority is the organization of early multidisciplinary care of nephropathies that are associated with diabetes or hypertension, which are presently the most frequent kidney diseases in all stages, including on renal replacement therapy. Such a model would need a lot of efforts and courageous political decisions, but this is probably the only way to go, for a long term advantage for the society. Nonetheless, the fragmentation of care, which often leads to the “fragmentation of patients”, is a threat against its development.

Increasingly, however, nephrologists are confronted with conflicting healthcare goals. They are expected to ensure prevention and early diagnosis, yet, at the same time, limits the core of their activities to selected nephropathies and advanced kidney diseases. This dilemma will be discussed in the next section.

### 1.6. Political and Economic Barriers to Prevention and Care

Budget constraints make it difficult to ensure prevention and early CKD care.

The high prevalence of CKD, an average of 10% of the world’s population, is the main barrier to extending renal care by nephrologists to all patients. Given the extreme heterogeneity of kidney diseases, it is not easy to find a satisfactory algorithm for identifying patients with potentially progressive diseases so that they can be referred to a specialist. As stated above, in most European countries over 30% of patients start dialysis in emergency and with suboptimal care and this high prevalence clearly shows the limits of present CKD care [26,36,37,38,39,40]. The example of the prevalence of dialysis incidence in the United States, which is almost twice as high as Canada’s (after correction for case-mix), is the best large-scale demonstration of the importance of pre-dialysis care, widely available in Canada, but sadly lacking in the U.S. [32]. When considering that, depending on setting, the expenditure for one to two years of in-hospital dialysis (calculated to cost between 50,000 and 80,000 Euros per year) is equivalent to a nephrologist’s yearly salary, the reasons for optimizing the nephrology taskforce are evident [74].

While not having an adequate number of specialists hinders the development of prevention programs in which the nephrologist’s activity is central, a further challenge is the shift from in-hospital to out-of-hospital care. Day-hospital treatment in Italy is limited to therapeutic procedures, thus leading to unnecessary hospitalization or patchwork care, which is so expensive that many patients cannot afford treatment. The situation is more favourable in France, where complex or multiple diagnostic procedures are allowed in day-hospital. However, in both France and Italy, out-of-hospital consultations are poorly reimbursed. The fee for a consultation, that will normally last at least 30 min, is 30 to 40 Euros; thus, an activity which requires high quality consultation is not “paying for itself”, and this is a major limit for developing out-patient care, in particular in small or private centres.

Dialysis is an expensive treatment; in most European countries the key determinant of cost of treatment is nurses’ and doctors’ salaries [9,10,11,24]. The more years of work experience a staff member has, the higher their salary will be, which means that “old” experts cost more than young beginners. At a time when we are increasingly dealing with a greying dialysis population, reducing nursing and medical staff negatively influences the quality of care that is provided, paradoxically penalizing the more specialised hospital settings, since patients with lower comorbidity are more efficiently treated out-of hospital.

While in settings, such as the Trentino region in Italy, in which a single public centre runs all of the satellite services and is responsible for the care of over 300 dialysis patients, internal adjustments serve to equilibrate resources, this may not be the case where “simpler” patients are followed by private dialysis units, determining a different case mix, which tends to be further enhanced by the development of home dialysis techniques, in particular, if they are self-managed.

### 1.7. Care for Kidney Disease or Care for Kidney Patients?

The difference between caring for kidney diseases and caring for kidney patients is not merely semantic, but one that influences the overall organization of care. For example, the size of a hospital’s nephrology wards determines the number of physicians working in each of its services. How patients on dialysis or with kidney transplantation are seen is a key factor in how treatment is organized: if the focus is on kidney diseases, only patients with dialysis-related problems or with complications of renal transplantation that affect the kidney will be hospitalized in a nephrology ward, while, for example, a dialysis or transplant patient with pneumonia will be sent to an infectious medicine or internal medicine ward. Conversely, if the focus is on patients and not on disease, all kidney patients that do not specifically require hospitalization in another specialist context, such as cardiology or surgery, will be hospitalized in nephrology.

While reducing the size of specialized services, which is the current trend, would favour the first choice, there are several good clinical and logistic reasons for defending the second one: continuity of care (kidney patients will already be known in nephrology, and the importance of homogeneous chronic care has been demonstrated); expertise in the management of drugs in kidney disease (a crucial point for all CKD patients; in Europe, drug interference is the most common cause of AKI in the in-hospital setting [75,76]); expertise which makes possible the timely definition of indications for acute and chronic dialysis (dialysis sessions may need to be tailored in acute illness in chronic dialysis patients; dialysis may be needed in case of graft failure [77]); psychological benefits when patients in a moment of stress are managed by people they know; a logistical advantage which derives from more direct access to a dialysis ward.

While, to the best of our knowledge, no scientific society has clearly set forth guidelines indicating which patients should be managed in a nephrology setting, some, including the French Society of Nephrology, have quantified the number of beds needed to ensure optimum care of dialysis patients in nephrology wards (one hospital bed reserved for each 40 patients on chronic dialysis) [31].

While a hypertrophic nephrology ward may not be favourable in terms of bed management, small services risk being non-competitive; to determine what the best policy to follow in hospitalizing kidney patients is, we need to perform a detailed analysis of factors, such as the duration of hospital stay and the risk of readmission of patients in nephrology versus non nephrology wards, and of patients’ preferences.

Independently from the size of the hospital wards, hospitalization for any reason should be seen as a precious occasion for diagnosis of chronic kidney diseases, an occasion that is, however, often lost [27].

### 1.8. Clinical Research: Can It Be the Key of a Resurgence of Clinical Nephrology?

Quantity is an issue in nephrology, in which a smaller or larger community is called to respond to the needs of a population, which remains out of proportion to the offer of care (at least 40% of the patients start dialysis without appropriate care, as previously discussed [28,29,36,37,38,39]); the risk of being overwhelmed by routine clinical work is obvious.

However, doing clinical research is a powerful means for remaining updated, linked with a network of experts. Hospital centres often have a larger catchment area than university ones, but they generally publish less clinical research. The contraposition between university and non-university hospital is sharper for example in France than in Italy. Interestingly, the French biometric incentive system, based upon a biometric tool called SIGAPS has failed to increase research in nephrology in non-university settings. The system attributes a score to every article published, which is based on the “position” of its authors and on the “rank” of the journal, and ultimately corresponds to a significant economic incentive to the hospital, as high as about 65,000 Euros, divided in four years, for one paper published as first of last name on a top 10% journal [78]. Lack of expertise in medical writing, language barriers, lack of incentives to services and physicians, lack of time to devote to non-clinical activities limit the impact of this highly rewarding system: a first or last name on a paper published in a journal in the second quartile, presently corresponds to the reimbursement of about 250 clinical consultations per year for four years, thus potentially allowing for a considerable re-investment of time.

Conversely, although clinical research is not economically rewarded in Italy, a relatively large number of research papers are published, and not all of those doing research have links to universities. The presence of a higher number of nephrologists in Italy s surely one factor, but the frequent lack of adequate infrastructure, and the limited availability of services (secretarial staff, informatics tools, etc.) negatively impacts on efficiency. The wider borders of the speciality, still oriented towards the “holistic” model and the need to defend an endangered specialty, in the context of an economic crisis, may have played a role in focusing attention on research in this latter country.

To cite a recent paper on the importance of cultivating a nephrology research workforce, “A healthy nephrology workforce is presented as the fruits of nurturing by all of the invested stakeholders. Although not all nephrologists will elect to be part of the research workforce, all require influential mentors to attract them to the discipline” [79].

## 2. Conclusions

Increased reflection and research on the planning of the specificities and models of nephrology care is urgently needed if we wish to rise to the challenge of providing earlier and better care for older and more complex kidney patients with acute and chronic kidney diseases, with reduced budgets.
